# An increase in environmental temperature within the growth range suppresses phage resistance in *Escherichia coli*

**DOI:** 10.1128/aem.01188-25

**Published:** 2026-01-30

**Authors:** Satoshi Takayama, Yoshimitsu Masuda, Ken-ichi Honjoh, Takahisa Miyamoto

**Affiliations:** 1 Laboratory of Food Hygienic Chemistry, Division of Food Science and Biotechnology, Department of Bioscience and Biotechnology, Faculty of Agriculture, Kyushu University 12923, Fukuoka, Japan; Anses, Maisons-Alfort Laboratory for Food Safety, Maisons-Alfort, France

**Keywords:** phage resistance, LPS truncation, thermosensitivity

## Abstract

To develop countermeasures against phage-resistant bacteria without antibiotics, a detailed phenotypic characterization of phage-resistant *Escherichia coli* BW25113 was performed. Phage susceptibility testing of *E. coli* BW25113 deletion mutants involved in lipopolysaccharide (LPS) synthesis revealed that the first glucose residue of the LPS outer core was essential for infection by phage S127. From *E. coli* BW25113 cells that survived S127 exposure, four phage-resistant strains were isolated and characterized. Sodium dodecyl sulfate-polyacrylamide gel electrophoresis analysis showed that the phage-resistant strains had LPS with a smaller molecular mass compared with that of the *E. coli* BW25113 parental strain. Fluorescence microscopy after *Bac*Light staining, along with comparisons of viable counts on non-selective versus selective media, indicated increased membrane permeability in the resistant strains, resulting in heightened sensitivity to monocaprin, a natural non-ionic surfactant. Furthermore, upon elevating the culture temperature from 30°C to 37°C, the resistant strains exhibited increased Congo Red binding and autoaggregation, which was not observed in the parental strain. Viability assays revealed that both the phage-resistant strains and deep rough mutants, such as *ΔhldE* and *ΔwaaG*, did not grow at 46°C. Notably, regrowth after phage S127 treatment at 37°C was significantly delayed in the *E. coli* BW25113 parental strain grown at 46°C than in that grown at 37°C. *E. coli* populations that became phage resistant owing to truncated LPS chains were highly sensitive to hydrophobic antibacterial substances and high temperatures, suggesting that these could be critical factors for controlling phage-resistant bacteria.

**IMPORTANCE:**

The application of phages in agriculture and food-producing environments often faces challenges in the control of phage-resistant bacteria. To effectively address this issue, a deeper understanding of the unique phenotypes associated with phage resistance is warranted. Few studies have suppressed the regrowth of phage-resistant populations without using antibiotics, based on detailed phenotypic characterization. Here, we report that the phage-resistant *Escherichia coli* population selected by lytic phage S127 was sensitive to elevated temperature and decreased viability at 46°C. Furthermore, Congo Red binding and autoaggregation, which have been reported to exhibit unique behaviors in *E. coli* deep rough mutants, were dependent on high culture temperature. Our findings highlight a novel, exploitable phenotype of phage resistance in host bacteria that could be applied to the biocontrol of phage resistance in foodborne pathogens without the use of antibiotics in practical settings.

## INTRODUCTION

The widespread use of conventional antimicrobial agents has led to the emergence of antimicrobial resistance (AMR), which is now recognized as a major global public health concern and a critical food safety issue (1, 2). A comprehensive global assessment of AMR in 2022 estimated that bacterial AMR was associated with 4.95 million deaths in 2019, with projections reaching 10 million deaths annually by 2050 if no effective countermeasures are implemented (3, 4). Any environment where antimicrobials are administered can be a risk factor for the selection and dissemination of AMR bacteria. In particular, the use of antimicrobials in animals/crops production facilitates the transmission of foodborne AMR bacteria to humans through the consumption of contaminated food or drinking water (5, 6). The foodborne AMR bacteria have made it challenging to treat patients with foodborne illness; thus, approaches to tackle AMR bacteria and means to prevent the emergence of AMR are in urgent need (7) throughout the process of “farm to folk” (8–10).

An alternative strategy to mitigate the emergence of AMR bacteria is the use of bacteriophages (phages) as antibacterial agents (11, 12). Phages are bacterial viruses that exist in every environment of the biosphere inhabited by host bacteria. Foods and food-producing environments are no exception to this inherent existence of phages. Exploiting their nature as bacterial predators, phages have been applied to control foodborne pathogens (13–15). From the perspective of AMR, the key advantages of phages are their ability to kill AMR bacteria, their sustainability as naturally abundant antimicrobial resources (16, 17), without contributing to the emergence or spread of AMR. Phages also offer distinct benefits as food additives. First, being naturally occurring, they do not alter the flavor or texture of foods, enabling effective decontamination without synthetic antimicrobials while maintaining the original quality of food or feed (14, 18, 19). Second, harmless microflora native to the environment may not be eliminated because phages selectively infect and lyse their target bacterium, which differentiates them from existing antimicrobials with broad spectra (20, 21). Taken together, these attributes highlight phages as promising antimicrobial agents that can be applied in multiple stages of the food chain.

Despite their potential, it is typically observed that a certain population of the host bacterium survives phage treatment and regrows. This is “phage resistance” of the host, escaping from infection by the same phage. For phages to be practically applied to control foodborne pathogens, this resistant population must be successfully suppressed. Several studies have explored the mechanisms of phage resistance (22), which are generally classified into three categories: modification of phage receptors, host phage defense systems, and phage-derived phage defense systems (23). Among these, receptor modification frequently alters cell-surface properties, leading to a reduction in stress tolerance or pathogenicity of phage-resistant bacteria (24, 25). This phenomenon highlights the trade-off associated with acquiring phage resistance (26). To date, numerous studies have attempted to exploit this trade-off between phage resistance and reduced stress tolerance to control phage-resistant populations, although they have often focused on the efficacy of phage-antibiotic combinations or decreased pathogenicity, particularly in clinical settings (27–30). Therefore, there have been few reports on control methods based on the characteristics of phage-resistant bacteria that do not use antibiotics. From the One Health perspective, it is crucial to explore the possibility of controlling phage resistance in a different way.

In the present study, we aimed to establish a scientific basis for developing novel strategies to control phage-resistant bacteria without the use of antibiotics by characterizing the unique phenotypes of phage-resistant strains. *Escherichia coli* and the lytic phage S127BCL3 (S127) were used as model organisms to investigate phage-host interactions and to analyze phage-resistant bacteria. Phage S127, a lytic phage of the genus *Vequintavirus*, was originally isolated from chicken livers using *E. coli* O157:H7 as an original host and possesses a 135.5 kbp genomic DNA (31).

Building on these findings, we discuss the thermosensitivity of phage-resistant strains, which leads to distinctive phenotypes in phage-resistant strains, and the possibility of controlling phage resistance by applying mild heat treatment to target bacterial populations.

## RESULTS

### The first glucose residue of lipopolysaccharide outer core for efficient infection by phage

**Fig 1:**
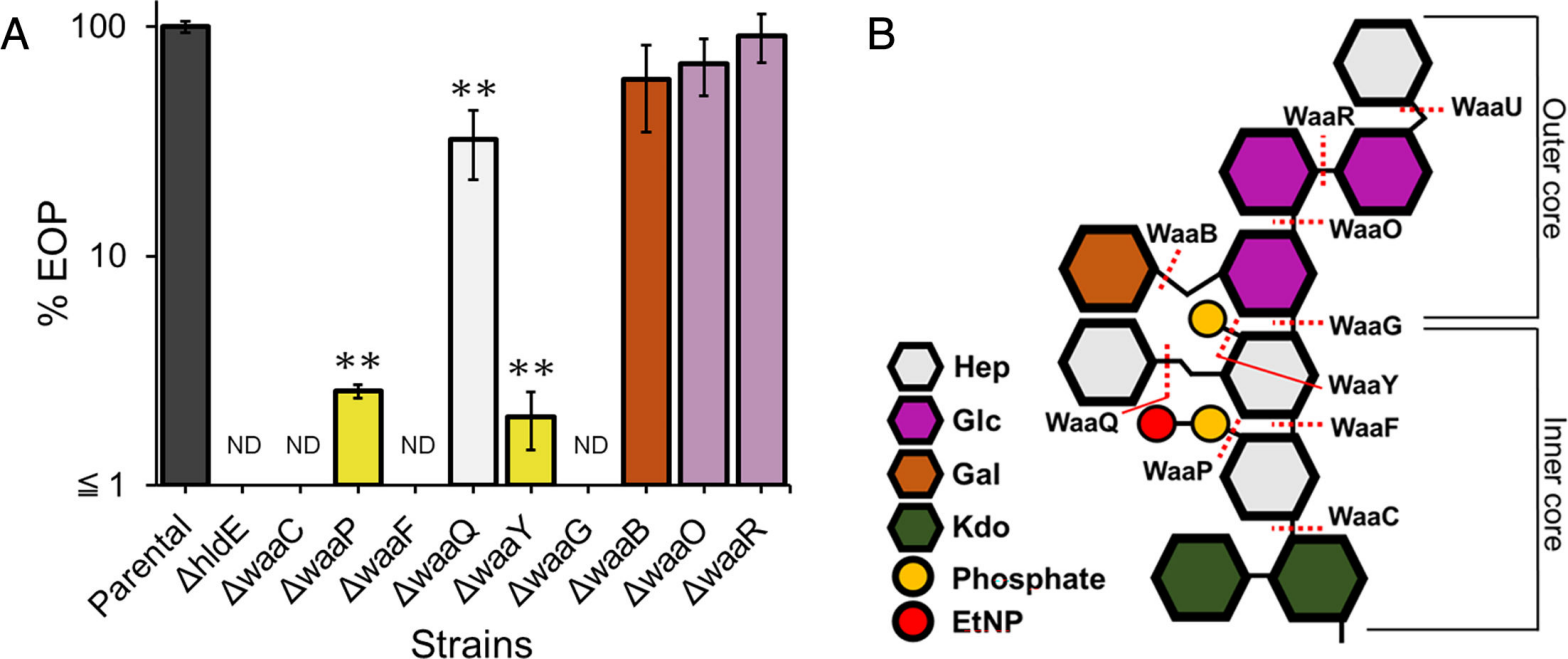
EOP of phage S127 on mutants with various lengths of LPS. (**A**) EOP of S127 on parental (black) and a series of single-gene deletion mutants lacking genes responsible for modifications of the LPS core region (other colors). Error bars show standard deviations of the mean for three biological replicates. The data were analyzed using one-way analysis of variance with Dunnett’s multiple comparison to parental. **P < 0.01. ND, not detected. (**B**) Structure of the LPS core from *E. coli* BW25113. Abbreviations are as follows: Hep, L-*glycero*-D-*manno*-heptose; Glc, D-glucose; Gal, D-galactose; Kdo, 3-deoxy-D-*mannno*-oct-2-ulosonic acid; P, phosphate; and EtNP, 2-aminoethyl phosphate. The assignment of function to genes encoding core glycosyltransferases and phosphotransferases has been previously reported (33, 34).

We identified the putative receptor required for phage S127 to infect *E. coli* BW25113. Since lipopolysaccharide (LPS) commonly serves as a receptor for phage infection in gram-negative bacteria (32), the efficiency of plating (EOP) of the phage was evaluated against a series of *E. coli* BW25113 mutants, each lacking a single gene involved in LPS synthesis. In several mutants, the EOP of S127 was significantly decreased (Fig. 1A). These included deletions in genes responsible for the modification of the LPS inner core region (*hldE*, *waaC*, *waaP*, *waaF*, *waaQ*, *waaY*, and *waaG*). The degree of LPS truncation was determined based on the role of each deleted gene product in LPS core biosynthesis, as illustrated in the structure of *E. coli* BW25113 (Fig. 1B). The *hldE* gene product, HldE, is involved in the biosynthesis of heptose in the inner core of LPS. Of these seven genes, the deletion of *hldE*, *waaC*, *waaF*, and *waaG* resulted in completely abolished S127 infection, whereas the deletion of *waaO* had little effect on EOP, suggesting that the first glucose residue of the LPS outer core was critical for S127 infection. Additionally, deletion of *waaP* and *waaY*, both of which are involved in phosphorylation of heptose residues in the LPS inner core, also caused a significant decrease in EOP. Collectively, these results indicated that the boundary region between the outer and inner core of LPS, as well as the phosphate modifications of heptose, could play a role in maintaining the surface structure for phage S127 to initiate its infection.

### Characterization of S127-resistant strains

**Fig 2:**
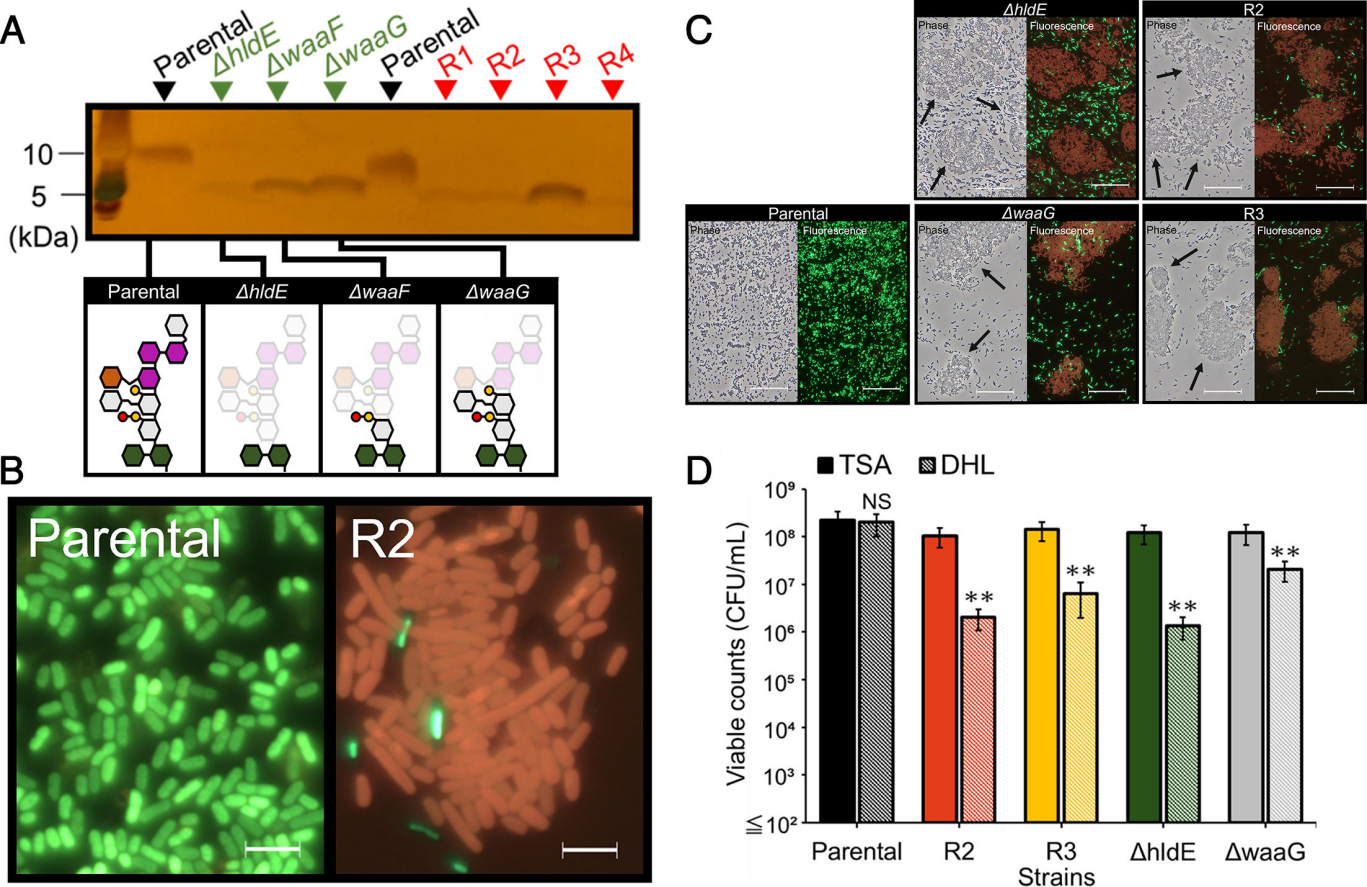
Characterization of phage-resistant strains of *E. coli* BW25113. (**A**) Silver staining of LPS extracted from phage-resistant strains. (**B**) *Bac*light staining of parental and a phage-resistant strain, R2, as representative images. Scale bars indicate 5 µm. (**C**) Wider view of the result of *Bac*light staining. Black arrows point out cellular aggregates observed in phage-resistant strains. Scale bars indicate 50 μm. (**D**) Comparison of viable cell counts of parental (black) and R2 (orange), R3 (yellow), *ΔhldE* (green), and *ΔwaaG* (gray) between tryptic soy agar (TSA; solid) and deoxycholate hydrogen sulfide lactose (DHL) agar (dashed). Error bars show standard deviations of the mean for three biological replicates. The data were analyzed using a two-tailed Student’s *t*-test. **P < 0.01. NS, not significant.

Spontaneous phage S127-resistant strains were isolated from single colonies formed by bacterial cells that survived overnight phage treatment in Luria-Bertani (LB) broth at a multiplicity of infection (MOI) of 0.1. The phage resistance of the four isolates (designated as R1, R2, R3, and R4) was confirmed by plaque assays, in which no plaques were detected on their lawns. To characterize the unique phenotypes of these strains, the LPS of the phage-resistant strains was analyzed. Sodium dodecyl sulfate-polyacrylamide gel electrophoresis analysis of purified LPS, visualized by silver staining, revealed that all phage-resistant strains produced LPS chains with a smaller molecular mass (MM) than that of the parental strain (Fig. 2A). The MM of LPS from R1, R2, and R4 was similar to that of *ΔhldE*, while that from R3 resembled *ΔwaaG*. Overall, the LPS profiles in the phage-resistant strains were comparable to those of *ΔhldE*, *ΔwaaF*, and *ΔwaaG* mutants, suggesting that phage-resistant strains possessed truncated LPS chains relative to the parental strain. To investigate the phenotypes of strains with distinct types of LPS, R2 and R3 were selected as representative strains for further characterization.

To investigate the effect of LPS truncation on membrane property, changes in the outer membrane permeability (35) of phage-resistant strains were assessed. Fluorescence microscopy following *Bac*Light staining revealed stronger red fluorescence derived from propidium iodide (PI) in phage-resistant strains such as R2, compared with the parental strain (Fig. 2B). Moreover, aggregates formed by PI-stained cells were frequently observed in the phage-resistant strains (Fig. 2C, black arrows). Viability assays on tryptic soy agar (TSA) and deoxycholate hydrogen sulfide lactose (DHL) agar plates also indicated that most of the cells of phage-resistant strains were viable but had an incomplete envelope function (Fig. 2D). These results suggested that the integrity of the outer membrane was compromised, leading to increased outer membrane permeability in the cells of phage-resistant strains, which exhibited enhanced aggregation.

The susceptibility of phage-resistant and mutant strains to monocaprin, a hydrophobic antimicrobial agent, was investigated to verify increased outer membrane permeability. As shown in Table 1, the phage-resistant strains exhibited increased susceptibility to monocaprin, with minimum inhibitory concentration (MIC) values decreasing to 1/8 in R2 and *ΔhldE* and 1/2 in R3 and *ΔwaaG* compared with that of *E. coli* BW25113 parental strain (Table 1).

**TABLE 1:** MIC of monocaprin^*a*^ for the parental strain and its derivatives

Strain	MIC (µM)
Parental (BW25113)	>2,000
R2	250
R3	1,000
*ΔhldE* (JW3024)	250
*ΔwaaG* (JW3606)	1,000

^*a*^ Each strain was cultured in the presence of monocaprin. MIC was defined as the lowest final concentration of monocaprin at which no bacterial growth was observed across all three biological replicates.

### Effects of culture temperature on curli production and autoaggregation

**Fig 3:**
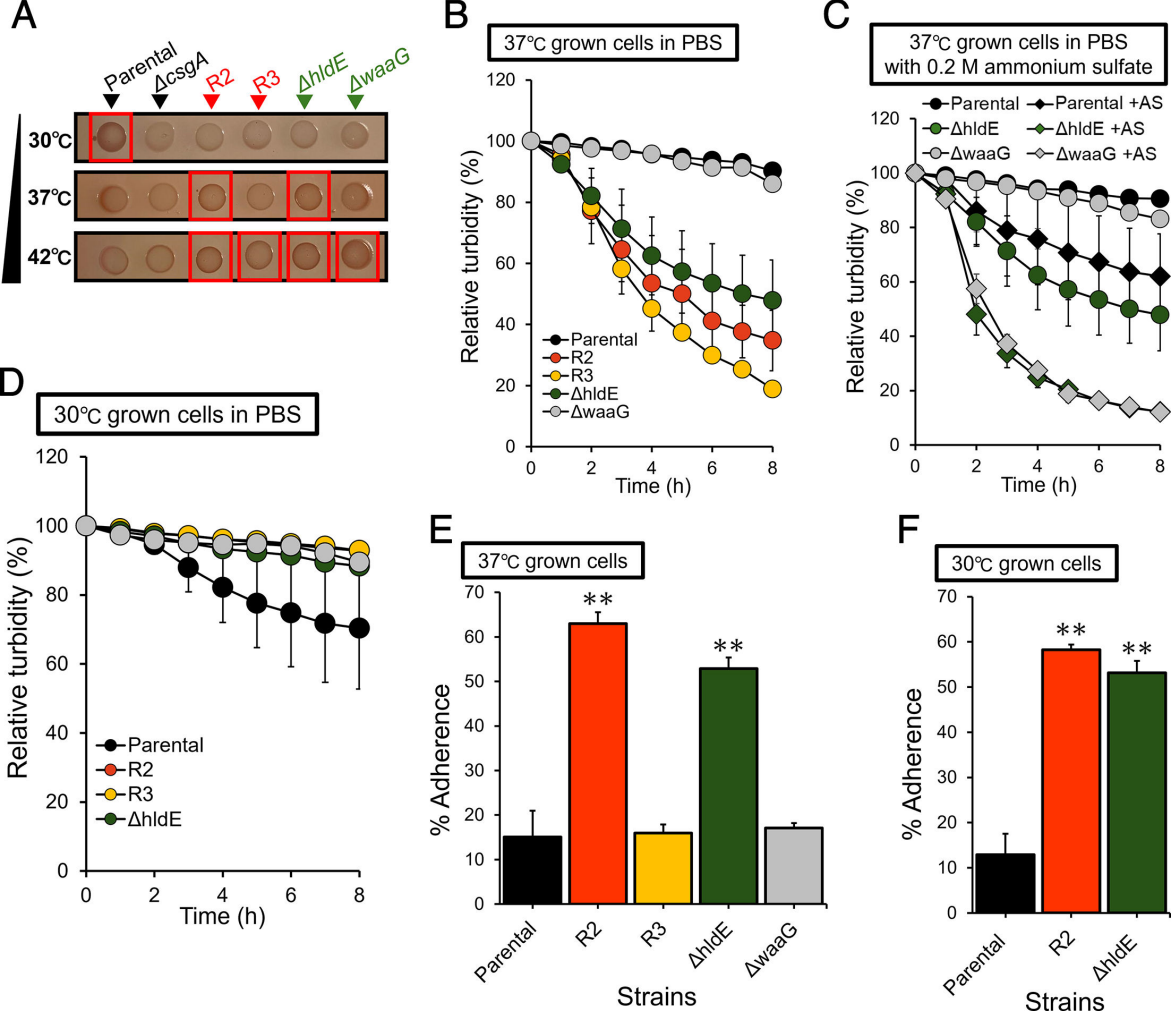
Effects of culture temperatures on curli fimbriae production, autoaggregation, and cell-surface hydrophobicity of *E. coli* strains. (**A**) CR binding of *E. coli* BW25113 parental, *ΔcsgA*, R2, R3, *ΔhldE*, and *ΔwaaG*. Each strain was cultured on a YESCA plate in the presence of CR at different temperatures. (**B**) Changes in OD_600_ of the cell suspensions in phosphate-buffered saline (PBS) of *E. coli* BW25113 parental (black), R2 (orange), R3 (yellow), *ΔhldE* (green), and *ΔwaaG* (gray) strains cultured at 37°C were determined. (**C**) Changes in OD_600_ of the cell suspensions in PBS supplemented with 0.2 M ammonium sulfate of *E. coli* BW25113 parental (black), *ΔhldE* (green), and *ΔwaaG* (gray) strains cultured at 37°C were determined. Ammonium sulfate-supplemented groups (+AS) are shown with diamonds. (**D**) Changes in OD_600_ of the cell suspensions in PBS of *E. coli* BW25113 parental (black), R2 (orange), R3 (yellow), *ΔhldE* (green), and *ΔwaaG* (gray) strains cultured at 30°C were determined. (**E**) Cell-surface hydrophobicity of parental (black), R2 (orange), R3 (yellow), and *ΔhldE* (green) cultured at 37°C or (**F**) 30°C determined by the degree of adherence of bacterial cells to *n-*octane. Error bars show standard deviations of the mean for three biological replicates, and data were analyzed using one-way analysis of variance followed by Dunnett’s multiple comparisons to parental. **P < 0.01.

The involvement of curli fimbriae in the autoaggregation of phage-resistant strains was examined. The red coloration of the colony formed by the parental strain on YESCA agar containing Congo Red (CR) decreased as the culture temperature increased (Fig. 3A). Pale white colonies were observed in *ΔcsgA*, which is deficient in the synthesis of curli fimbriae, indicating the dependency of red colony phenotype on curli production. The red coloration of the colony by phage-resistant strains, as well as *ΔhldE* and *ΔwaaG*, increased at 37°C or 42°C and decreased at 30°C. This observation was the opposite of that observed in the parental strain, where curli production was prominent at a lower temperature.

In the autoaggregation assay, the isolated phage-resistant strains and *ΔhldE* cultured at 37°C showed a rapid decrease in turbidity of bacterial suspensions in phosphate-buffered saline (PBS), whereas the parental strain and *ΔwaaG* did not (Fig. 3B). The addition of ammonium sulfate to PBS promoted a decrease in turbidity of bacterial suspensions from *ΔhldE* and *ΔwaaG* (Fig. 3C), indicating that autoaggregation in these strains was salt concentration dependent. Autoaggregation was canceled in all bacterial cell suspensions prepared from the cells cultured at 30°C (Fig. 3D), which was consistent with the temperature dependency observed in the CR binding of phage-resistant strains (Fig. 3A).

To examine the involvement of hydrophobic interactions in the autoaggregation of phage-resistant cells, cell-surface hydrophobicity was assessed using the bacterial adhesion to hydrocarbons (BATH) assay, which measures adherence of bacterial cells to *n-*octane. When cultured at 37°C, the percent adherence of R2 and *ΔhldE* strains was significantly higher than that of the parental, R3, and *ΔwaaG* strains (Fig. 3E). To evaluate the temperature dependency of the cell-surface hydrophobicity, parental, R2, and *ΔhldE* strains were also cultured at 30°C. Cells of R2 and *ΔhldE* strains showed higher percent adherence than that of the parental strain (Fig. 3F), indicating that these strains consistently exhibited higher cell-surface hydrophobicity than the parental strain, irrespective of culture temperature.

### Effects of culture temperature on the growth of phage resistance of the isolated strains and deletion mutants

**Fig 4:**
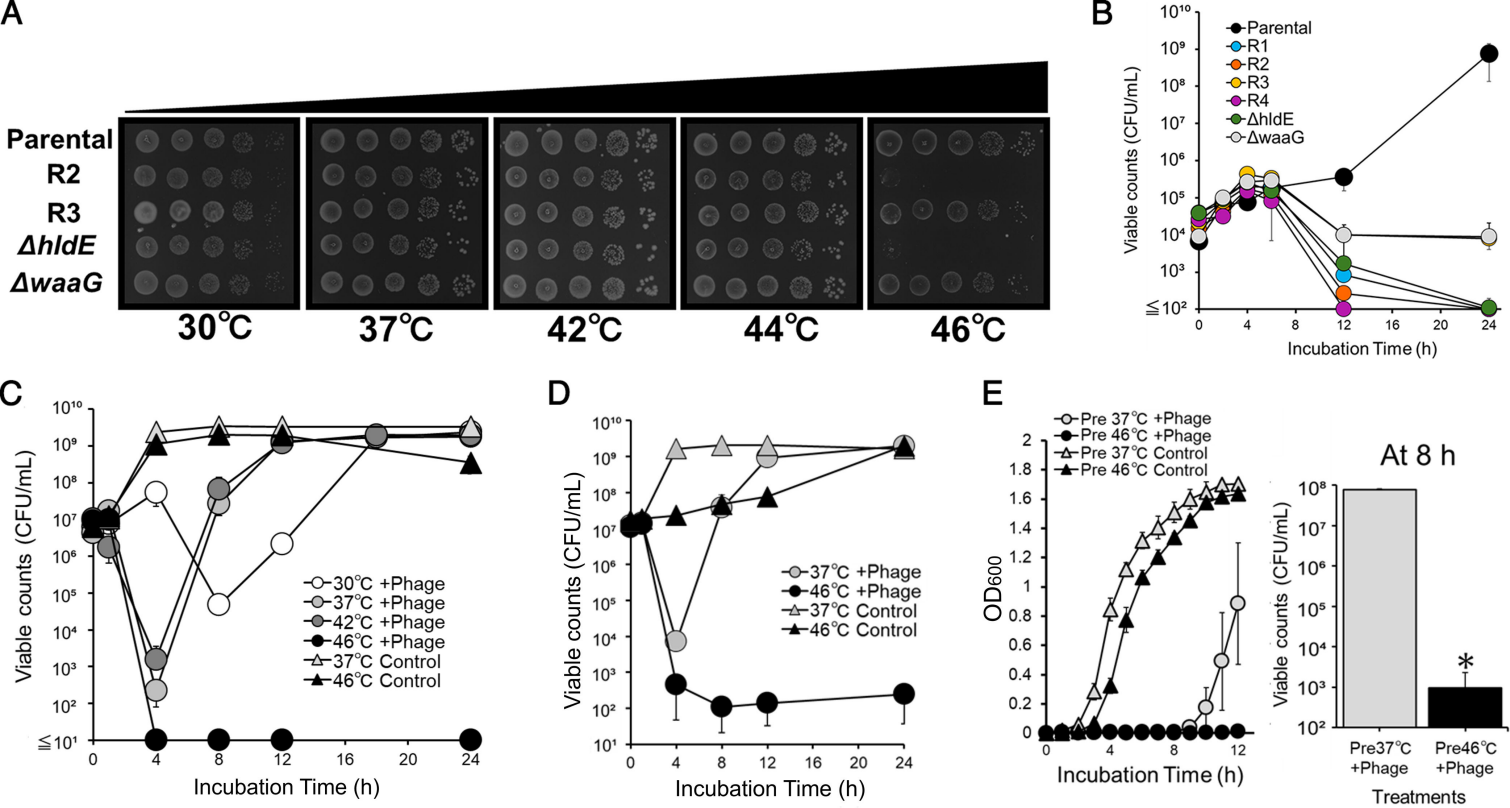
Effects of culture temperatures on regrowth and viability of *E. coli* strains in the absence and presence of phage S127. (**A**) Colony formation of *E. coli* BW25113 parental and R2, R3, *ΔhldE*, and *ΔwaaG* strains was examined on LB agar plates at 30°C, 37°C, 42°C, 44°C, and 46°C. (**B**) Changes in viability at 46°C in LB broth of parental (black), R1 (blue), R2 (orange), R3 (yellow), R4 (purple), *ΔhldE* (green), and *ΔwaaG* (gray) strains precultured at 37°C. (**C**) *E. coli* BW25113 parental strain was incubated with phage S127 (circles) at 30°C (white), 37°C (light gray), 42°C (dark gray), and 46°C (black) or without phage at 37°C and 46°C (triangles) at MOI = 0.1. (**D**) EHEC strain *E. coli* No. 127 was incubated with phage (circles) at 37°C (light gray) and 46°C (black) or without phage (triangles). (**E**) Effect of preculture at 46°C on the susceptibility to phage S127 in BW25113 parental strain. *E. coli* BW25113 parental strain was precultured at 37°C (light gray) or 46°C (black) and then incubated with phage (circles) or without phage (triangles) at MOI = 100. The OD600 of the culture was measured at designated time points (left graph), and viable cell counts were measured after 8 h of incubation (right). Error bars show the standard deviation of the mean for three biological replicates. Data were analyzed using the Mann-Whitney *U* test for the comparison of two treatments at 8 h. *P < 0.05.

To examine the thermosensitivity of the phage-resistant and mutant strains, their colony-forming abilities were determined at different culture temperatures on LB agar. Deep rough mutants, including *ΔhldE* and *ΔwaaG*, were examined in this study owing to their inability to grow at 46°C (36). All strains grew on LB agar at 44°C; however, only parental, R3, and *ΔwaaG* strains grew at 46°C, whereas R2 and *ΔhldE* did not (Fig. 4A). A time-kill assay in LB broth at 46°C further supported these observations. All strains increased their viable counts for the first 4 h, but thereafter, all except the parental strain showed a decline. By 24 h, the viable counts of R1, R2, R4, and *ΔhldE* decreased to the detection limit, whereas R3 and *ΔwaaG*, which retained LPS inner core structures, still maintained 10^4^ CFU/mL (Fig. 4B). Parental cells grown at 37°C were treated with phage S127 at different temperatures. In the presence of the phage, viable counts initially decreased but eventually recovered along with regrowth of the phage-resistant population at 30°C, 37°C, and 42°C. In contrast, at 46°C, viable counts dropped to the detection limit within 4 h and showed no recovery for 20 h at 46°C (Fig. 4C). A similar effect was observed in EHEC strain *E. coli* No. 127, where the phage-resistant population grew significantly slower when treated with phage S127 at 46°C (Fig. 4D). Finally, the effect of the incubation temperature before phage treatment on the survival of phage-resistant populations was investigated. To this end, the parental strain precultured at 37°C or 46°C was treated with phage S127 at 37°C. Regrowth of *E. coli* BW25113 precultured at 46°C was significantly delayed in the presence of phage compared with that of the cells precultured at 37°C, as shown by reduced turbidity (Fig. 4E, left). After 8 h of phage treatment, viable cell counts differed by 10^5^-fold between the cells precultured at 37°C and 46°C (Fig. 4E, right).

## DISCUSSION

Bacterial phage resistance associated with phenotypic trade-offs has been of growing interest because it is valuable for optimizing bacterial control strategies (25, 37). In this study, *E. coli* isolates resistant to phage S127 were characterized to develop novel strategies for controlling phage-resistant populations without antibiotics. The requirement of the first glucose residue in the LPS outer core for S127 infection (Fig. 1A), together with the lower MM of the LPS chain in all isolated phage-resistant strains (Fig. 2A), indicated that deletion of the outer core of the LPS chain conferred resistance to S127 in *E. coli* BW25113. Fluorescence microscopy and viability assays on TSA and DHL agar further demonstrated that the truncation of the LPS chain compromised membrane integrity, resulting in an increase in viable cells with increased outer membrane permeability (Fig. 2B through D). Since the LPS core oligosaccharide portion confers hydrophilicity to the cell surface (38), the truncation of the LPS chain resulted in increased cell-surface hydrophobicity in R2 and *ΔhldE* strains (Fig. 3E) and heightened their susceptibility to monocaprin (Table 1). Monocaprin, a non-ionic surfactant and fatty acid monoglyceride, is generally recognized as safe and widely used in food as both an emulsifier and antimicrobial (39–41). These findings suggest that further evaluation of monocaprin’s efficacy against phage-resistant bacteria is warranted. The susceptibility to monocaprin and cell-surface hydrophobicity of the R2 closely resembled those of *ΔhldE*, whereas R3 resembled *ΔwaaG* (Table 1; Fig. 3E). Consistent with this phenotypic variation, the strains also differed in autoaggregation and thermosensitivity. Collectively, the degree of LPS truncation produced distinct phenotypic outcomes among the phage-resistant isolates (Fig. 3A through D and 4B). The physiological mechanisms underlying these phenotypes, along with the potential applications of increased thermosensitivity in phage-resistant bacteria, are discussed below.

Autoaggregation is a hallmark of *E. coli* deep rough mutants (38, 42). In the present study, autoaggregation was investigated in relation to potential mediators, including curli fimbriae, cell-surface hydrophobicity, environmental salt concentration, and culture temperature. Previous studies on curli production in deep rough mutants have yielded conflicting conclusions, likely due to differences in culture temperatures (43, 44). The CR binding assay suggested that curli production in the phage-resistant strains increased at higher culture temperatures, in contrast to the parental strain, where curli expression was greater at lower temperatures (Fig. 3A). Because curli expression is regulated by σ^S^ (45), which can be indirectly induced by σ^E^ (46), it is plausible that the shorter LPS chains in resistant strains trigger envelope stress at elevated temperatures, thereby increasing σ^E^ activity and, in turn, σ^S^-dependent curli expression. In line with these CR binding patterns, autoaggregation in phage-resistant strains was also culture temperature dependent. However, the BATH assay indicated that the inherent increase in cell-surface hydrophobicity in R2 and *ΔhldE* did not correlate with autoaggregation behavior (Fig. 3E and F). The R3 strain, which likely retains an inner core heptose in its LPS, exhibited strong autoaggregation despite having hydrophobicity levels similar to the parental strain (Fig. 3B). These findings suggest that factors beyond hydrophobicity are involved in autoaggregation. Ag43 and Type I fimbriae, encoded by *flu* and *fimH*, respectively, are well-known mediators of autoaggregation in *E. coli* (47, 48), whereas Nakao *et al*. have shown that even a double deletion of *flu* and *hldE* did not abolish enhanced autoaggregation in *E. coli* BW25113 (38). Similarly, overnight cultures of *ΔcsgA* and *ΔfimH* treated with phage S127 still showed a rapid decrease in turbidity (data not shown), indicating that loss of single aggregation-mediating factors is insufficient to abolish autoaggregation in deep rough populations. For *ΔwaaG*, previous studies reported that autoaggregation was comparable to the parental strain when cells were suspended in PBS (38), whereas enhanced autoaggregation of *ΔwaaG* was observed when cells were suspended in LB broth (42, 49), which contains a larger amount of organic compounds compared with PBS, suggesting that autoaggregation of *ΔwaaG* depends on the substances in the cell suspension. Based on these facts, it was shown in this study that the autoaggregation of *ΔwaaG* and *ΔhldE* appeared to be solute concentration dependent (Fig. 3C). Taken together, these results highlight the multifactorial nature of autoaggregation, which is influenced by multiple factors, including culture temperature and salt concentration. Given the diversity of curli production and autoaggregation with changes in culture temperature in phage-resistant strains, further analysis is warranted to elucidate the underlying mechanisms and potential impact of these behaviors on the outcome of phage therapy.

In the present study, the elevated temperature dependence of CR binding and autoaggregation in phage-resistant strains prompted a detailed investigation of their thermosensitivity. Membrane integrity has been reported to play a critical role in bacterial thermotolerance (50, 51). Reduced membrane integrity can induce oxidative stress caused by the disruption of the electron transport chain and impair the ability to maintain cell structure due to increased membrane fluidity, which affects adaptation to external temperature changes (51, 52). Intact LPS structure has been reported to maintain outer membrane integrity by enabling divalent cations to crosslink adjacent LPS chains via phosphate groups and by contributing to the balanced composition of phospholipids and proteins in the outer membrane (35, 53, 54). Consistent with the increased membrane permeability observed in phage-resistant strains (Fig. 2B through D), the truncation of the LPS chain likely reduces membrane stability by compromising these critical envelop factors (55, 56), which impaired their thermotolerance, resulting in a decreased acclimatization ability and growth suppression at 46°C (Fig. 4A and B). Previous studies have shown that the extent of LPS chain truncation resulted in the difference in outer membrane properties in *E. coli* (38, 57), and the difference in thermosensitivity among the strains investigated here could be explained by the varied magnitude of destabilization of the outer membrane in each strain, which corresponded with their susceptibility to monocaprin (Fig. 2D). Specifically, R2 and *ΔhldE*, which had a lower MIC value for monocaprin, were more thermosensitive than R3 and *ΔwaaG*. Increased thermosensitivity in phage-resistant bacteria may also occur in other gram-negative bacteria infected by phages that require LPS as a receptor, similar to S127.

In addition to suppressing the growth of phage-resistant strains by elevating the culture temperature (Fig. 4C and D), we observed increased susceptibility to phage S127 in the parental *E. coli* BW25113 when elevating the preculture temperature (Fig. 4E). Based on previous reports on phenotypic heterogeneity in bacterial populations (58, 59), cells harboring truncated LPS chains may naturally occur within the parental population of *E. coli*, representing potential phage-resistant subpopulations that display increased thermosensitivity. Accordingly, preculture at 46°C may have reduced the subpopulation of these thermosensitive, potential phage-resistant cells, thereby explaining the delayed regrowth of phage-resistant cells following phage treatment of the parental cells precultured at 46°C. Alternatively, the observed effect may be partly attributable to impaired adaptation of cells shifted from elevated to lower temperatures, as indicated by the prolonged lag phase of the parental strain precultured at 46°C (Fig. 4E, left). As the experiment was performed based on the hypothesis that potential phage-resistant cells preexist prior to phage treatment, further analysis to confirm their presence in the parental population is required to better understand this phenomenon. Nevertheless, the results suggest that a high-temperature history within the normal growth range significantly decreases the population of phage-resistant cells, leading to delayed regrowth of phage-resistant bacteria.

Although it remains to be confirmed whether the increased thermosensitivity of *E. coli* phage-resistant strains demonstrated in this study is also observed in other gram-negative bacteria, exploring the feasibility of applying mild heat treatment combined with phage treatment is worthwhile. Such an approach could be particularly useful for controlling phage-resistant bacterial populations in food-manufacturing environments as well as veterinary and clinical settings. For example, maintaining food at elevated temperatures above 46°C after phage treatment may prevent potential regrowth of the phage-resistant population of the target bacterium, while the growth of off-target bacteria should be taken into account at the same time. In addition, the combined use of phage therapy and hyperthermia could be evaluated as a therapeutic option for wounds resulting from bacterial infections in the veterinary and clinical contexts. Further elucidation to ascertain the practical exploitation of increased thermosensitivity is anticipated.

## MATERIALS AND METHODS

### Bacterial strains, phage, and culture conditions

*E. coli* BW25113 parental and single-gene knockout mutants from the Keio collection were obtained from the National BioResource Project (NBRP, Shizuoka, Japan). *E. coli* O157: H7 strain No. 127 was kindly provided by the Fukuoka City Institute of Health and Environment (Fukuoka, Japan). *E. coli* strains were cultured in LB Miller broth (Kanto Chemical, Japan) or on LB agar plates at 37°C unless otherwise specified. The BW25113 single-gene knockout mutants used in this study included *ΔhldE* (JW3024), *ΔwaaC* (JW3596), *ΔwaaP* (JW3605), *ΔwaaF* (JW3595), *ΔwaaQ* (JW3607), *ΔwaaY* (JW3600), *ΔwaaG* (JW3606), *ΔwaaB* (JW3603), *ΔwaaO* (JW3602), *ΔwaaR* (JW3601), and *ΔcsgA* (JW1025). Each mutant harbored a kanamycin-resistant cassette, as previously described (60), and was precultured in LB containing 20 µg/mL kanamycin. Phage S127BCL3 (S127) was originally isolated from chicken liver in a previous study, and the phage S127 solution was prepared as described elsewhere (31).

### Isolation of phage-resistant strains

Phage-resistant strains were isolated from bacterial cultures that were treated overnight with phage S127. Briefly, an overnight culture of *E. coli* BW25113 parental strain was diluted with LB broth to get a 10⁸ CFU/mL suspension. Fifty microliter of phage S127 solution was added to 4.95 mL of the diluted *E. coli* suspension in a test tube to attain a MOI of 0.1. The mixture in the test tube was incubated overnight at 37°C with shaking. The resulting culture was then streaked onto LB agar plates, and four single colonies that formed on the agar were randomly selected. Each colony was purified by two successive re-streakings on a fresh LB agar plate, each accompanied by cultivation in LB broth. The phage-resistant phenotype was confirmed by plaque assays, in which no plaque formation was observed after spotting the phage solution onto bacterial lawns of the isolates. Finally, the purified resistant cultures were stored in 20% glycerol at −80°C for subsequent characterization.

### EOP test

EOP of phage S127 was evaluated against the *E. coli* BW25113 parental strain and its single-gene deletion derivatives with various lengths of the LPS core. Ten microliter of serially diluted phage solutions was spotted onto bacterial lawns prepared from each strain and incubated overnight at 37°C. The phage titer against each strain was calculated based on the plaque numbers. The EOP was determined by dividing the phage titer obtained for each mutant strain by that of the parental strain.

### LPS extraction and silver staining

LPS was extracted from overnight bacterial cultures using the hot aqueous phenol extraction (61). Purified LPS was separated by sodium dodecyl sulfate-polyacrylamide gel electrophoresis on 1.2% acrylamide gel containing 4 M urea. Silver staining (62) was used to visualize the LPS bands in the gel.

### Membrane permeability assay

The membrane permeability of the test strains was assessed using fluorescence microscopy with *Bac*Light Live/Dead Bacterial Viability Kit (Invitrogen). Overnight cultures were diluted approximately 1:100 in 3 mL of fresh LB broth in a test tube and incubated at 37°C until the OD_600_ reached 0.4. Cells were then collected by centrifugation at 8,000 × *g*, 4°C for 5 min, washed twice with 0.1 M HEPES buffer (pH 7.1), and suspended in 100 µL of the same buffer. Staining was performed following the manufacturer’s instructions, except that the bacterial suspension was incubated for 2 h. Microscopic observation was performed using a BX53 fluorescence microscope (OLYMPUS) equipped with a U-FBW dichroic mirror (OLYMPUS), which provided excitation at 460–495 nm and emission at 510 nm or higher. In this assay, intact cells appear green due to SYTO9 staining, whereas cells with increased membrane permeability are stained red as they allow the penetration of PI.

### Counting viable cells and cells with incomplete envelope function

To quantify cells with incomplete envelope function, viable and intact cells were assessed using spot tests on TSA and DHL agar with modifications to previously described methods (63, 64). Bacterial suspensions of each strain in 0.1 M HEPES buffer (pH 7.1) were prepared as described above for the membrane permeability assay and serially diluted in PBS. Ten microliter of each diluent was spotted onto both TSA (Oxoid, UK) and DHL agar (Nissui Pharmaceutical, Japan) plates. After overnight incubation at 37°C, the colonies were enumerated. Because sodium deoxycholate in DHL agar inhibits the growth of cells with incomplete envelope function, only intact cells form colonies on DHL agar, whereas both intact cells and envelope-compromised cells form colonies on TSA. The difference in the viable counts between TSA and DHL agar thus indicates the number of viable cells with incomplete envelope function.

### MIC measurements

The MIC of monocaprin was determined by evaluating the growth of the test strains in the presence of various concentrations of monocaprin. Monocaprin (Taiyo Kagaku, Japan) was dissolved in 100% dimethyl sulfoxide and serially diluted in LB broth containing 1% dimethyl sulfoxide in 96-well plates. For inoculum preparation, overnight cultures were adjusted to an OD_600_ = 0.1 in LB broth and further diluted 1:100. Equal volumes (100 µL) of bacterial suspension were added to wells containing 100 µL of the broth with varying monocaprin concentrations. The plates were incubated at 37°C for 24 h with shaking, and OD_600_ of each well was measured using a spectrophotometer (Infinite F50, TECAN) after the incubation. The MIC of monocaprin against different strains was defined as the lowest concentration at which the OD_600_ of the well remained less than 0.01 after 24 h of cultivation.

### BATH assay

Cell-surface hydrophobicity was assessed by examining bacterial adherence to *n*-octane using the BATH assay (65) with some modifications. Briefly, cells were suspended in potassium phosphate buffer containing 3.5 M ammonium sulfate (pH 7.1) to attain an OD_600_ of 1.3–1.4 using a spectrophotometer (UV-1800, SHIMADZU). Two hundred microliters of *n*-octane (Nacalai Tesque, Japan) was added to 1.5 mL of the cell suspension in a test tube (φ 10 mm), followed by incubation at 30°C for 10 min. The suspension was then vigorously vortexed for 2 min and incubated at room temperature for 20 min to allow phase separation. The bottom aqueous phase was transferred to a 1 mL disposable cuvette (Dsp Semi 1.5 ML, Fisher Scientific) using a Pasteur pipette, and the OD_600_ of the aqueous phase was measured. Adherence to *n-*octane was expressed as the percentage decrease in OD_600_ after vortexing, relative to the initial OD_600_ of the aqueous phase before vortexing, using the following formula: percent adherence = [1 – (OD_600_ after vortex/OD_600_ before vortex)] × 100.

### CR binding assay

CR is an anionic diazo dye that binds specifically to amyloid fibers such as curli fimbriae in *E. coli* and to polysaccharides such as cellulose and chitin (66). CR binding was evaluated as previously described, with minor modifications (67). Overnight cultures of test strains were adjusted to equal turbidity and were spotted onto YESCA agar containing CR (1% casamino acids, 0.1% yeast extract, 2% agar, 20 µg/mL CR [SIGMA], and 10 µg/mL Coomassie Brilliant Blue R-250 [Nacalai Tesque]). The plates were incubated statically overnight at 30°C, 37°C, and 42°C for 24 h. CR binding was assessed based on the intensity of the red coloration of colonies after incubation. Note that the cellulose production by *E. coli* BW25113 was abolished owing to the mutation of *bcsQ*, which is involved in cellulose synthesis (68). Accordingly, the red color of the colony can be attributed to curli production in BW25113 (69).

### Autoaggregation assay

The autoaggregation assay was performed by measuring the reduction in turbidity of bacterial cell suspensions due to cell precipitation, as previously described (38, 48). Briefly, overnight-cultured cells were harvested by centrifugation at 6,000 × *g*, 25°C for 5 min, and the resulting pellets were resuspended in PBS (pH 7.4) to an OD_600_ of 1.2–1.4. The suspensions were then statically incubated at room temperature in the test tubes. When required, ammonium sulfate (Nacalai Tesque) was supplemented to PBS to a final concentration of 0.2 M. At designated time points, the OD_600_ of the phase above the sediment formed by aggregation was measured using a spectrophotometer (Miniphoto 518R, TAITEC). Autoaggregation was expressed as relative turbidity, defined as the ratio of turbidity at a given time point to the initial turbidity.

### Thermosensitivity test

The thermosensitivity of the strains was evaluated using viability assays at different temperatures. Overnight cultures grown at 37°C of test strains were diluted in LB broth to an OD_600_ = 0.1 and serially diluted in PBS, and 10 µL of each dilution was spotted onto LB agar. Plates were incubated overnight at 30°C, 37°C, 42°C, 44°C, and 46°C, and colony formation was assessed. A time-kill assay was also performed in a broth at 46°C. Briefly, overnight cultures of the test strains were diluted in 5 mL of LB broth and incubated with shaking at designated temperatures. At each specified time point, 20 µL of an aliquot was taken and serially diluted in PBS, and 10 µL of each dilution was spotted on LB agar. Viable cell counts were determined based on the number of colonies formed on the plate after incubation for 24 h at 37°C and the dilution factor.

### Phage treatment at different temperatures

*E. coli* BW25113 parental strain and *E. coli* O157:H7 No. 127, an EHEC strain, were used as test strains. Phage-treated bacterial cultures were prepared as described previously. For the control, the same volume of SM buffer (50 μL) was added in place of the phage solution. The cultures were incubated with or without phage S127 at different temperatures with shaking, and viable cell counts were determined at designated time points using a spot test. For undiluted samples, 100 µL of culture was directly plated onto LB agar.

### Phage treatment of bacteria with different temperature histories

The effect of a high-temperature history prior to phage treatment was evaluated using the *E. coli* BW25113 parental strain precultured at different temperatures. Briefly, 10 µL of an overnight culture of parental strain was transferred into 5 mL of fresh LB broth and incubated overnight at either 37°C or 46°C. The cultures were then diluted to an OD_600_ = 0.1, and 50 µL of the diluted suspension was inoculated into 4.95 mL of LB containing S127 at 10^8^ PFU/mL, yielding approximately 100 of MOI. Following incubation at 37°C, OD_600_ was measured at designated time points using a spectrophotometer (Miniphoto 518R, TAITEC), and viable counts were determined using the spot test or direct plating.

### Statistical analysis

All statistical analyses were performed using Statcel4 (70) implemented in Microsoft Excel. Specific details regarding the statistical methods are provided in the corresponding figure legends.

## Data Availability

The data used to support the findings are included in the article. Further raw data can be available upon request.

## References

[1] Okeke IN, de Kraker MEA, Van Boeckel TP, Kumar CK, Schmitt H, Gales AC, Bertagnolio S, Sharland M, Laxminarayan R. 2024. The scope of the antimicrobial resistance challenge. Lancet 403:2426–2438. doi:10.1016/S0140-6736(24)00876-6 38797176

[2] Djordjevic SP, Jarocki VM, Seemann T, Cummins ML, Watt AE, Drigo B, Wyrsch ER, Reid CJ, Donner E, Howden BP. 2024. Genomic surveillance for antimicrobial resistance — a One Health perspective. Nat Rev Genet 25:142–157. doi:10.1038/s41576-023-00649-y 37749210

[3] O’Neill J. 2016. Tackling drug-resistant infections globally: final report and recommendations. The Review on Antimicrobial Resistance, chaired by Jim O’Neill. London HM Government and Wellcome Trust

[4] Murray CJL, Ikuta KS, Sharara F, Swetschinski L, Robles Aguilar G, Gray A, Han C, Bisignano C, Rao P, Wool E, et al.. 2022. Global burden of bacterial antimicrobial resistance in 2019: a systematic analysis. Lancet 399:629–655. doi:10.1016/S0140-6736(21)02724-0 35065702 PMC8841637

[5] FAO, WHO. 2023. Foodborne antimicrobial resistance – Compendium of Codex standards. First revision. Codex Alimentarius Commission, Rome.

[6] Kniel KE, Kumar D, Thakur S. 2018. Understanding the complexities of food safety using a “one health” approach. Microbiol Spectr 6. doi:10.1128/microbiolspec.pfs-0021-2017 PMC11633553 29451115

[7] Ghosh C, Sarkar P, Issa R, Haldar J. 2019. Alternatives to conventional antibiotics in the era of antimicrobial resistance. Trends Microbiol 27:323–338. doi:10.1016/j.tim.2018.12.010 30683453

[8] Allen HK, Levine UY, Looft T, Bandrick M, Casey TA. 2013. Treatment, promotion, commotion: antibiotic alternatives in food-producing animals. Trends Microbiol 21:114–119. doi:10.1016/j.tim.2012.11.001 23473629

[9] FAO, VMD. 2022. Tackling antimicrobial use and resistance in food-producing animals – Lessons learned in the United Kingdom. Rome.

[10] EU. 2020. Farm to Fork Strategy – For a fair, healthy and environmentally-friendly food system. Brussels. https://food.ec.europa.eu/document/download/472acca8-7f7b-4171-98b0-ed76720d68d3_en?filename=f2f_action-plan_2020_strategy-info_en.pdf.

[11] Gordillo Altamirano FL, Barr JJ. 2019. Phage therapy in the postantibiotic era. Clin Microbiol Rev 32:e00066-18. doi:10.1128/CMR.00066-18 30651225 PMC6431132

[12] Strathdee SA, Hatfull GF, Mutalik VK, Schooley RT. 2023. Phage therapy: from biological mechanisms to future directions. Cell 186:17–31. doi:10.1016/j.cell.2022.11.017 36608652 PMC9827498

[13] Colavecchio A, Goodridge LD. 2017. Phage therapy approaches to reducing pathogen persistence and transmission in animal production environments: opportunities and Challenges. Microbiol Spectr 5. doi:10.1128/microbiolspec.pfs-0017-2017 PMC11687507 28664828

[14] Endersen L, Coffey A. 2020. The use of bacteriophages for food safety. Curr Opin Food Sci 36:1–8. doi:10.1016/j.cofs.2020.10.006

[15] Ranveer SA, Dasriya V, Ahmad MF, Dhillon HS, Samtiya M, Shama E, Anand T, Dhewa T, Chaudhary V, Chaudhary P, Behare P, Ram C, Puniya DV, Khedkar GD, Raposo A, Han H, Puniya AK. 2024. Positive and negative aspects of bacteriophages and their immense role in the food chain. NPJ Sci Food 8:1. doi:10.1038/s41538-023-00245-8 38172179 PMC10764738

[16] Chibani-Chennoufi S, Bruttin A, Dillmann ML, Brüssow H. 2004. Phage-host interaction: an ecological perspective. J Bacteriol 186:3677–3686. doi:10.1128/JB.186.12.3677-3686.2004 15175280 PMC419959

[17] Dion MB, Oechslin F, Moineau S. 2020. Phage diversity, genomics and phylogeny. Nat Rev Microbiol 18:125–138. doi:10.1038/s41579-019-0311-5 32015529

[18] Endersen L, O’Mahony J, Hill C, Ross RP, McAuliffe O, Coffey A. 2014. Phage therapy in the food industry. Annu Rev Food Sci Technol 5:327–349. doi:10.1146/annurev-food-030713-092415 24422588

[19] Lewis R, Hill C. 2020. Overcoming barriers to phage application in food and feed. Curr Opin Biotechnol 61:38–44. doi:10.1016/j.copbio.2019.09.018 31726332

[20] Greer GG. 2005. Bacteriophage control of foodborne bacteria. J Food Prot 68:1102–1111. doi:10.4315/0362-028x-68.5.1102 15895751

[21] Abuladze T, Li M, Menetrez MY, Dean T, Senecal A, Sulakvelidze A. 2008. Bacteriophages reduce experimental contamination of hard surfaces, tomato, spinach, broccoli, and ground beef by Escherichia coli O157:H7. Appl Environ Microbiol 74:6230–6238. doi:10.1128/AEM.01465-08 18723643 PMC2570303

[22] Hampton HG, Watson BNJ, Fineran PC. 2020. The arms race between bacteria and their phage foes. Nature 577:327–336. doi:10.1038/s41586-019-1894-8 31942051

[23] Egido JE, Costa AR, Aparicio-Maldonado C, Haas PJ, Brouns SJJ. 2022. Mechanisms and clinical importance of bacteriophage resistance. FEMS Microbiol Rev 46:fuab048. doi:10.1093/femsre/fuab048 34558600 PMC8829019

[24] Kortright KE, Chan BK, Koff JL, Turner PE. 2019. Phage therapy: a renewed approach to combat antibiotic-resistant bacteria. Cell Host Microbe 25:219–232. doi:10.1016/j.chom.2019.01.014 30763536

[25] Mangalea MR, Duerkop BA. 2020. Fitness trade-offs resulting from bacteriophage resistance potentiate synergistic antibacterial strategies. Infect Immun 88:e00926-19. doi:10.1128/IAI.00926-19 32094257 PMC7309606

[26] Ferenci T. 2016. Trade-off mechanisms shaping the diversity of bacteria. Trends Microbiol 24:209–223. doi:10.1016/j.tim.2015.11.009 26705697

[27] Oromí-Bosch A, Antani JD, Turner PE. 2023. Developing phage therapy that overcomes the evolution of bacterial resistance. Annu Rev Virol 10:503–524. doi:10.1146/annurev-virology-012423-110530 37268007

[28] Kortright KE, Done RE, Chan BK, Souza V, Turner PE. 2022. Selection for phage resistance reduces virulence of Shigella flexneri. Appl Environ Microbiol 88:e0151421. doi:10.1128/AEM.01514-21 34788068 PMC8788674

[29] León M, Bastías R. 2015. Virulence reduction in bacteriophage resistant bacteria. Front Microbiol 6:343. doi:10.3389/fmicb.2015.00343 25954266 PMC4407575

[30] Shen Y, Loessner MJ. 2021. Beyond antibacterials – exploring bacteriophages as antivirulence agents. Curr Opin Biotechnol 68:166–173. doi:10.1016/j.copbio.2020.11.004 33333352

[31] Lin CY, Murayama T, Futada K, Tanaka S, Masuda Y, Honjoh KI, Miyamoto T. 2024. Screening of genes involved in phage-resistance of Escherichia coli and effects of substances interacting with primosomal protein A on the resistant bacteria. J Appl Microbiol 135:lxad318. doi:10.1093/jambio/lxad318 38142224

[32] Bertozzi Silva J, Storms Z, Sauvageau D. 2016. Host receptors for bacteriophage adsorption. FEMS Microbiol Lett 363:fnw002. doi:10.1093/femsle/fnw002 26755501

[33] Heinrichs DE, Yethon JA, Whitfield C. 1998. Molecular basis for structural diversity in the core regions of the lipopolysaccharides of Escherichia coli and Salmonella enterica. Mol Microbiol 30:221–232. doi:10.1046/j.1365-2958.1998.01063.x 9791168

[34] Klein G, Müller-Loennies S, Lindner B, Kobylak N, Brade H, Raina S. 2013. Molecular and structural basis of inner core lipopolysaccharide alterations in Escherichia coli: incorporation of glucuronic acid and phosphoethanolamine in the heptose region. J Biol Chem 288:8111–8127. doi:10.1074/jbc.M112.445981 23372159 PMC3605630

[35] Nikaido H. 2003. Molecular basis of bacterial outer membrane permeability revisited. Microbiol Mol Biol Rev 67:593–656. doi:10.1128/MMBR.67.4.593-656.2003 14665678 PMC309051

[36] Murata M, Fujimoto H, Nishimura K, Charoensuk K, Nagamitsu H, Raina S, Kosaka T, Oshima T, Ogasawara N, Yamada M. 2011. Molecular strategy for survival at a critical high temperature in Eschierichia coli. PLoS One 6:e20063. doi:10.1371/journal.pone.0020063 21695201 PMC3112155

[37] Barber OW, Miramontes IM, Jain M, Ozer EA, Hartmann EM. 2021. The future of bacteriophage therapy will promote antimicrobial susceptibility. mSystems 6:e0021821. doi:10.1128/mSystems.00218-21 34282933 PMC8407298

[38] Nakao R, Ramstedt M, Wai SN, Uhlin BE. 2012. Enhanced biofilm formation by Escherichia coli LPS mutants defective in Hep biosynthesis. PLoS One 7:e51241. doi:10.1371/journal.pone.0051241 23284671 PMC3532297

[39] Thormar H, Hilmarsson H, Bergsson G. 2006. Stable concentrated emulsions of the 1-monoglyceride of capric acid (monocaprin) with microbicidal activities against the food-borne bacteria Campylobacter jejuni, Salmonella spp., and Escherichia coli. Appl Environ Microbiol 72:522–526. doi:10.1128/AEM.72.1.522-526.2006 16391087 PMC1352223

[40] Hyldgaard M, Sutherland DS, Sundh M, Mygind T, Meyer RL. 2012. Antimicrobial mechanism of monocaprylate. Appl Environ Microbiol 78:2957–2965. doi:10.1128/AEM.07224-11 22344642 PMC3318790

[41] U.S. Food and Drug Administration. 2011. Code of Federal Regulations. 21(1)B, part 184-B, section 184.1505:547. Washington, D.C U.S. Government Printing Office

[42] Laekas-Hameder M, Daigle F. 2024. Only time will tell: lipopolysaccharide glycoform and biofilm-formation kinetics in Salmonella species and Escherichia coli. J Bacteriol 206:e0031824. doi:10.1128/jb.00318-24 39315775 PMC11500611

[43] Smith DR, Price JE, Burby PE, Blanco LP, Chamberlain J, Chapman MR. 2017. The production of curli amyloid fibers is deeply integrated into the biology of Escherichia coli. Biomolecules 7:75. doi:10.3390/biom7040075 29088115 PMC5745457

[44] Maigaard Hermansen GM, Boysen A, Krogh TJ, Nawrocki A, Jelsbak L, Møller-Jensen J. 2018. HldE is important for virulence phenotypes in enterotoxigenic Escherichia coli. Front Cell Infect Microbiol 8:253. doi:10.3389/fcimb.2018.00253 30131942 PMC6090259

[45] Gualdi L, Tagliabue L, Landini P. 2007. Biofilm formation-gene expression relay system in Escherichia coli: modulation of σ^s^-dependent gene expression by the CsgD regulatory protein via σ^s^ protein stabilization. J Bacteriol 189:8034–8043. doi:10.1128/JB.00900-07 17873038 PMC2168689

[46] Fang FC. 2005. Sigma cascades in prokaryotic regulatory networks. Proc Natl Acad Sci USA 102:4933–4934. doi:10.1073/pnas.0501417102 15795367 PMC555993

[47] Schembri MA, Hjerrild L, Gjermansen M, Klemm P. 2003. Differential expression of the Escherichia coli autoaggregation factor antigen 43. J Bacteriol 185:2236–2242. doi:10.1128/JB.185.7.2236-2242.2003 12644494 PMC151503

[48] Schembri MA, Christiansen G, Klemm P. 2001. FimH-mediated autoaggregation of Escherichia coli. Mol Microbiol 41:1419–1430. doi:10.1046/j.1365-2958.2001.02613.x 11580845

[49] Wang Z, Wang J, Ren G, Li Y, Wang X. 2015. Influence of core oligosaccharide of lipopolysaccharide to outer membrane behavior of Escherichia coli. Mar Drugs 13:3325–3339. doi:10.3390/md13063325 26023839 PMC4483631

[50] Moon S, Ham S, Jeong J, Ku H, Kim H, Lee C. 2023. Temperature matters: bacterial response to temperature change. J Microbiol 61:343–357. doi:10.1007/s12275-023-00031-x 37010795

[51] Li H, Gänzle M. 2016. Some like it hot: heat resistance of Escherichia coli in food. Front Microbiol 7:1763. doi:10.3389/fmicb.2016.01763 27857712 PMC5093140

[52] Murata M, Ishii A, Fujimoto H, Nishimura K, Kosaka T, Mori H, Yamada M. 2018. Update of thermotolerant genes essential for survival at a critical high temperature in Escherichia coli. PLoS One 13:e0189487. doi:10.1371/journal.pone.0189487 29485997 PMC5828445

[53] Sun J, Rutherford ST, Silhavy TJ, Huang KC. 2022. Physical properties of the bacterial outer membrane. Nat Rev Microbiol 20:236–248. doi:10.1038/s41579-021-00638-0 34732874 PMC8934262

[54] Maher C, Hassan KA. 2023. The Gram-negative permeability barrier: tipping the balance of the in and the out. mBio 14:e0120523. doi:10.1128/mbio.01205-23 37861328 PMC10746187

[55] Yethon JA, Vinogradov E, Perry MB, Whitfield C. 2000. Mutation of the lipopolysaccharide core glycosyltransferase encoded by waaG destabilizes the outer membrane of Escherichia coli by interfering with core phosphorylation. J Bacteriol 182:5620–5623. doi:10.1128/JB.182.19.5620-5623.2000 10986272 PMC111012

[56] Smit J, Kamio Y, Nikaido H. 1975. Outer membrane of Salmonella typhimurium: chemical analysis and freeze-fracture studies with lipopolysaccharide mutants. J Bacteriol 124:942–958. doi:10.1128/jb.124.2.942-958.1975 1102538 PMC235985

[57] Pagnout C, Sohm B, Razafitianamaharavo A, Caillet C, Offroy M, Leduc M, Gendre H, Jomini S, Beaussart A, Bauda P, Duval JFL. 2019. Pleiotropic effects of rfa-gene mutations on Escherichia coli envelope properties. Sci Rep 9:9696. doi:10.1038/s41598-019-46100-3 31273247 PMC6609704

[58] Davis KM, Isberg RR. 2016. Defining heterogeneity within bacterial populations via single cell approaches. Bioessays 38:782–790. doi:10.1002/bies.201500121 27273675

[59] Beaumont HJE, Gallie J, Kost C, Ferguson GC, Rainey PB. 2009. Experimental evolution of bet hedging. Nature 462:90–93. doi:10.1038/nature08504 19890329

[60] Baba T, Ara T, Hasegawa M, Takai Y, Okumura Y, Baba M, Datsenko KA, Tomita M, Wanner BL, Mori H. 2006. Construction of Escherichia coli K-12 in-frame, single-gene knockout mutants: the Keio collection. Mol Syst Biol 2:2006.0008. doi:10.1038/msb4100050 PMC1681482 16738554

[61] Davis MR, Goldberg JB. 2012. Purification and visualization of lipopolysaccharide from Gram-negative bacteria by hot aqueous-phenol extraction. J Vis Exp 63:e3916. doi:10.3791/3916 PMC3466933 22688346

[62] Tsai C-M, Frasch CE, Sammons DW, Adams LD. 1982. A sensitive silver stain for detecting lipopolysaccharides in polyacrylamide gels. Anal Biochem 119:115–119. doi:10.1016/0003-2697(82)90673-x 6176137

[63] Masuda Y, Sakamoto E, Honjoh K-I, Miyamoto T. 2020. Role of toxin-antitoxin-regulated persister population and indole in bacterial heat tolerance. Appl Environ Microbiol 86:e00935-20. doi:10.1128/AEM.00935-20 32503909 PMC7414958

[64] Kobayashi H, Miyamoto T, Hashimoto Y, Kiriki M, Motomatsu A, Honjoh K-I, Iio M. 2005. Identification of factors involved in recovery of heat-injured Salmonella Enteritidis. J Food Prot 68:932–941. doi:10.4315/0362-028x-68.5.932 15895724

[65] Rosenberg M. 1984. Ammonium sulphate enhances adherence of Escherichia coli J-5 to hydrocarbon and polystyrene. FEMS Microbiol Lett 25:41–45. doi:10.1111/j.1574-6968.1984.tb01372.x

[66] Thongsomboon W, Werby SH, Cegelski L. 2020. Evaluation of phosphoethanolamine cellulose production among bacterial communities using Congo red fluorescence. J Bacteriol 202:e00030-20. doi:10.1128/JB.00030-20 32312746 PMC7283597

[67] Bordeau V, Felden B. 2014. Curli synthesis and biofilm formation in enteric bacteria are controlled by a dynamic small RNA module made up of a pseudoknot assisted by an RNA chaperone. Nucleic Acids Res 42:4682–4696. doi:10.1093/nar/gku098 24489123 PMC3985669

[68] Serra DO, Richter AM, Hengge R. 2013. Cellulose as an architectural element in spatially structured Escherichia coli biofilms. J Bacteriol 195:5540–5554. doi:10.1128/JB.00946-13 24097954 PMC3889604

[69] Arita-Morioka KI, Yamanaka K, Mizunoe Y, Tanaka Y, Ogura T, Sugimoto S. 2018. Inhibitory effects of Myricetin derivatives on curli-dependent biofilm formation in Escherichia coli. Sci Rep 8:8452. doi:10.1038/s41598-018-26748-z 29855532 PMC5981455

[70] Yanai H. 2015. Statcel: the useful add-in software forms on Excel, 4th ed. OMS.

